# Down-regulation of miR-138 promotes colorectal cancer metastasis via directly targeting TWIST2

**DOI:** 10.1186/1479-5876-11-275

**Published:** 2013-10-30

**Authors:** Limin Long, Guoqing Huang, Hongyi Zhu, Yonghong Guo, Youshuo Liu, Jirong Huo

**Affiliations:** 1 Department of Geriatrics, The Second Xiangya Hospital, Central South University, Changsha, Hunan 410011, PR China; 2 Department of Emergency, Xiangya Hospital, Central South University, Changsha, Hunan 410008, PR China; 3 Department of Gastroenterology, The Second Xiangya Hospital, Central South University, Changsha, Hunan 410011, PR China

**Keywords:** Colorectal cancer, Metastasis, Biomarker, MicroRNA138

## Abstract

**Background:**

Colorectal cancer (CRC) is the most common digestive system malignancy. The molecular events involved in the development and progression of CRC remain unclear. Recently, more and more evidences have showed that deregulated miRNAs participate in colorectal carcinogenesis.

**Methods:**

The expression levels of miR-138 were first examined in CRC cell lines and tumor tissues by real-time PCR. The *in vitro* and *in vivo* functional effects of miR-138 were examined further. Luciferase reporter assays were conducted to confirm the targeting associations. Kaplan-Meier analysis and log-rank tests were performed to estimate the overall survival and disease free survival rate.

**Results:**

miR-138 was found to be down-regulated in human colorectal cancer tissues and cell lines. Ectopic expression of miR-138 resulted in a dramatic inhibition of CRC migration and invasion *in vitro* and *in vivo*. Twist basic helix-loop-helix transcription factor 2 gene (TWIST2) was identified as one of the functional target. Restoration of miR-138 resulted in a dramatic reduction of the expression of TWIST2 at both mRNA and protein levels by directly targeting its 3′-untranslated region (3′UTR). Up-regulation of TWIST2 was detected in CRC tumors compared with adjacent normal tissues (P < 0.001) and is inversely correlated with miR-138 expression. We also identified that down-regulation of miR-138 was associated with lymph node metastasis, distant metastasis, and always predicted poor prognosis.

**Conclusion:**

These data highlight a pivotal role for miR-138 in the regulation of CRC metastasis by targeting TWIST2, and suggest a potential application of miR-138 in prognosis prediction and CRC treatment.

## Introduction

Colorectal cancer (CRC) is one of the most common digestive system tumors with high morbidity and mortality [1]. Recent progress in diagnosis and therapy methods has helped to cure this disease of many patients at early stages, but the prognosis for patients with advanced disease or metastasis is still very poor. Therefore, further investigations on the molecular pathogenesis of CRC and finding effective biomarkers were needed to distinguish patients with or without high metastatic risk followed by appropriate therapy. A series of studies had revealed that microRNAs (miRNAs) can regulate the expression of a variety of genes pivotal for tumor development and play important roles in the process of cancer migration and invasion [2-4].

miRNAs are 21- to 23-nucleotide, endogenous noncoding RNAs that negatively regulate target gene expressions by binding to its 3′UTR regions, leading to mRNA degradation or inhibiting translation into protein. miRNAs were reported to be associated with the pathogenesis of many human cancers [5], such as miR-124 with hepatocellular carcinoma cell [6], miR-30e* with human glioma [7], miR-125b with breast cancer [8], and so on. This result also applies to CRC, a subset of miRNAs was found to be aberrantly expressed in CRC [9-11]. However, the particular molecular mechanisms through which miRNAs mediate colorectal carcinogenesis and metastasis are still largely unknown. So the identification and characterization of the role of novel miRNAs in CRC may provide new insight into understanding the molecular mechanisms of CRC development. Based on Koji Okamoto et al. report, miR-138 was reduced expressed in the metastases in their experimental models and others found that it may be involved in ovarian cancer and non-small cell lung cancer metastasis [11-13], which suggested that miR-138 might play an important role in CRC progression.

In this study, we first detected the expressions of miR-138 in CRC tissues and cell lines, and a series of *in vitro* and *in vivo* studies were then conducted to investigate the mechanisms and impact of miR-138 in CRC. By bioinformatics analysis (TargetScan and MiRanda) and experimentally validation, we found TWIST2 was a direct target of miR-138 and miR-138 can inhibit CRC metastasis by targeting TWIST2. Finally, we find reduced expression miR-138 is usually associated with poor prognosis with CRC patients by statistical analysis.

## Materials and methods

### Cell culture

The human colorectal cell lines LoVo, DLD1, HCT116, SW620, HT29 and SW116 were obtained from the American Type Culture Collection (Manassas, VA, USA). HT29 were cultured in McCoy’s 5A medium (Invitrogen; Life Technologies, Carlsbad, CA, USA), LoVo, HCT116, SW620 and SW116 were cultured in RPMI 1640 containing 10% fetal bovine serum (Sigma-Aldrich, St. Louis, MO, USA), and DLD1 were grown in Dulbecco’s modified Eagle’s medium (DMEM)/F12 (Gibico, USA) supplemented with 10% fetal bovine serum (Sigma-Aldrich, St. Louis, MO, USA). All the cells were cultured in a humidified 37°C incubator supplemented with 5% CO_2_.

### Human samples

Paired resected surgical specimens from primary tumors were obtained from CRC patients who underwent initial surgery at The Second Xiangya Hospital, Changsha, according to a standard protocol, before any therapeutic intervention. The adjacent normal tissues at least 6 cm distant from the tumor were also taken. The eight normal tissues are the adjacent normal tissues from the 36 patients group (Stage I: one case; Stage II: three cases; Stage III: three cases; Stage IV: one cases). Following excision, tissue samples were immediately snap-frozen in liquid nitrogen and stored at -80°C until RNA extraction. The remaining tissue specimens were fixed in 10% formalin and embedded in paraffin for routine histological examination and IHC analysis. All subjects provided informed consent before specimen collection. The study protocol was approved by the Ethics Committee of The Second Xiangya Hospital of Central South University.

### RNA isolation and quantitative real-time PCR

Total RNA from cells and tissues was isolated using TRIzol reagent (Invitrogen, Carlsbad, California, USA) and was eluted in 50 μL nuclease free water. RNA concentration was measured by Nanodrop 2000 (Thermo Fisher Scientific, Wilmington, DE, USA). Real-time PCR were performed using the TaqMan MicroRNA Assay Kit (Applied Biosystems). Real-time PCR was performed using the Applied Biosystems 7900 Fast Real-Time PCR system (Applied Biosystems, Foster City, California, USA). The comparative cycle threshold (Ct) method was used to calculate the relative abundance of miRNA compared with RNA U6 (RNU6B) expression (fold difference relative to RNU6B).

### Western blot analysis

Total cellular protein was extracted and protein concentration was measured by the Bradford DC protein assay (Bio-Rad, Hercules, CA, USA). Then, a total of 50 μg protein was separated by SDS–PAGE and transferred to a polyvinylidene fluoride (PVDF) membrane. Membranes were incubated with primary antibodies. Primary antibodies included as follows: Twist2 (1:1,000, CST, USA) β-action (1:2,000, Santa Cruz Biotechnology, USA). The secondary antibody was a goat anti-mouse antibody (1:2000, Santa Cruz Biotechnology, USA). Proteins were visualized using the ECL procedure (Amersham Biosciences, USA). All experiments were performed three times.

### Colony formation assay

Cells (1 × 10^5^/well) were plated in a 24-well plate and transfected with miR-138 or control microRNA at 20 nmol/L by using lipofectamine 2000 (Invitrogen; Life Technologies). After 48 h, the cells were collected and seeded (500–1,000/ well) in a fresh six-well plates. After 12 days, visible colonies were fixed and stained with crystal violet.

### Vector construction and dual-luciferase reporter assay

For luciferase assays, the potential miR-138 binding site in the TWIST2 3′UTR was predicted at position 375–382 from the stop codon site of TWIST2 by TargetScan (http://www.targetscan.org) and miRanda (http://www.microRNA.org). Sequence of the 50-bp segment with the wild-type or mutant seed region was synthesized and cloned into the *Xba* I site of a pGL3-Control vector (Promega, USA) downstream from the luciferase stop codon; the new vectors were designated pGL3-TWIST2-WT and pGL3-TWIST2-MUT, respectively. The 293 T cells (1.5 × 10^5^ cells/well) were seeded in a 24-well plate and co-transfected 18 h later with pGL3-Control (0.4 mg) or pGL3-TWIST2-WT (0.4 mg) or pGL3-TWIST2-MUT (0.4 mg), pGL4.73 vector (a control Renilla luciferase vector, 50 ng, Promega, USA), and miR-138 (20 nM) or control microRNA (20 nM) using Lipofectamine 2000 (Invitrogen, USA). Cells were harvested 48 h after transfection, and luciferase activities were analyzed by the Dual-Luciferase Reporter Assay System (Promega, USA).

### In vivo assays

Female athymic BABL/c nude mice (4–6 weeks old) were purchased from the Experimental Animal Center of Guangdong Province (Guangzhou, China). One million HCT116-miR138 or HCT116-control stable cells were injected into the lateral tail veins of each nude mouse at a density of 1.0 × 10^7^ cells/ml. The animals were sacrificed, and their lungs were dissected and paraffin embedded after 11 weeks. Transverse sections (4–6 μm) of the lung were stained with hematoxylin and eosin (HE). Metastatic nodules were counted in a double-blind manner under microscopy, as described previously [14]. The animal studies and the experimental protocol were conducted in accordance with the current Chinese regulations and standards on the use of laboratory animals.

### Lentivirus packaging and stable cell line establishment

miR-138 and control microRNA precursor sequences were amplified from human genomic DNA and cloned into the *Bam*H I and *Mfe* I site of the lentiviral vector pEZX-MR03 (GeneCopoeiaTM) (pLV-miR-138, pLV-miR-control). Viruses were packaged in 293 T cells. Lenti-Pac HIV Expression Packaging Kit and pLV-miR-138 (or control) were co-transfected using EndoFectin Lenti transfection reagent following the manufacturer’s instruction (GeneCopoeiaTM). 293 T cells were cultured in DMEM with 5% FBS in a 37°C incubator with 5% CO_2_. Forty-eight hours after transfection, the supernatant was harvested, filtered and cleared by centrifugation at 500 × g for 10 min. Three days after infection, 2 μg/ml puromycin was added to the culture media to select the cell populations infected with the lentivirus for 2 weeks. The cell line expressing microRNA-138 stably was named HCT116-miR138; the control vector cell line was named HCT116-control. The expression of miR-138 was detected by real-time PCR in these two cell lines as described above.

### Cell migration and invasion assays

Cell migration and invasion activity was assessed by using a specialized Chamber (BD Biosciences) with or without BD BioCoat Matrigel according to the manufacturer’s instructions. Cells (5 × 10^4^ cells/mL) were loaded into chamber inserts containing an 8-μm pore size membrane with or without a thin layer matrigel matrix. Cells migrating to the lower surface of the membrane during 24 or 48 h were fixed with 100% methanol and non-invading cells on the upper surface of the membrane were removed. The membranes, with migrated cells, were then stained with crystal violet, scanned, and digital images were obtained with a microscope. The number of migrated and invasive cells was then determined for 5 independent fields under a microscope. The mean of triplicate assays for each experimental condition was used for analysis.

### Statistical analysis

Data were expressed as mean ± standard deviation (SD). The statistical significance of the studies was analyzed using Student’s *t* test (two-tailed). To conduct survival analysis, we included all of the CRC patients in a Kaplan-Meier analysis. A log rank test was used to compare different survival curves. All statistical analyses were performed using SPSS 13.0 software package. The probability of *P* < 0.05 was considered to be statistically significant.

## Results

### miR-138 was down-regulated in CRC cell lines and primary CRC tissues

**Figure 1:**
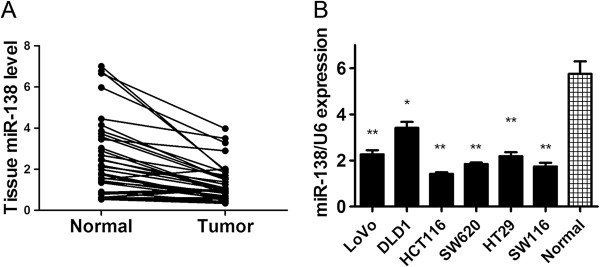
**miR-138 is commonly down-regulated in primary colorectal cancer tissues and colon cancer cell lines. (A)** Levels of miR-138 in 36 primary colorectal tumors compared with their adjacent normal tissues. The miR-138 level is normalized to RNU6B, and the *P* value indicated a significant difference in miR-138 level between paired samples. **(B)** The expression level of miR-138 in eight colon cancer cell lines and normal colon tissues (n = 8) by quantitative RT-PCR with RNU6B as an internal control. *P < 0.05, **P < 0.01.

We first examined the expression level of miR-138 in 36 human CRC tumor samples and paired normal colorectal tissues. Consistent with our exception, there was a 2–3 fold decrease in the expression level of miR-138 in the 36 tumors than in their paired normal tissues (*P* < 0.001)(Figure 1A). Among these paired samples, 86.1% (31/36) of the CRC samples showed lower miR-138 levels than in the adjacent normal tissues (see Figure 1A). We also examined miR-138 expression level in six CRC cell lines (LoVo, DLD1, HCT116, SW620, HT29, SW116) and observed that miR-138 was down-regulated in all six CRC cell lines relative to the normal primary colon cells from eight normal tissues (Figure 1B).

### Overexpression of miR-138 inhibited CRC cell colony formation, migration and invasion in vitro

**Figure 2:**
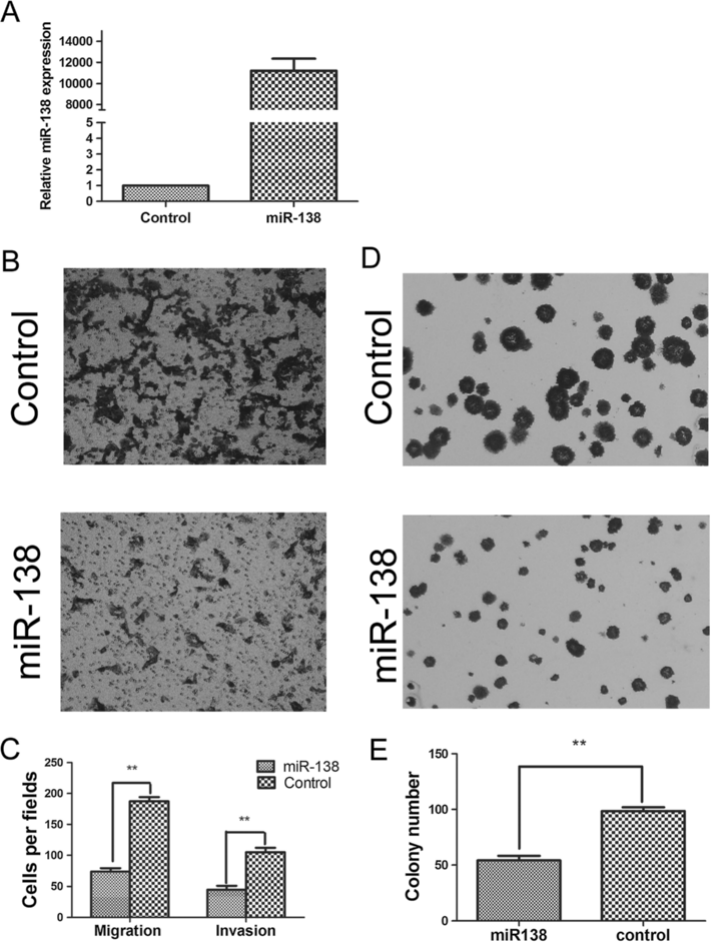
**Effect of miR-138 on migration, invasion and colony formation in CRC cell lines. (A)** Overexpression of miR-138 was demonstrated in HCT116 cells by real-time PCR. ***P < 0.001 **(B and C)** Overexpression of miR-138 can inhibit the migration and invasion of CRC cell lines. Representative transwell results for control-transfected and miR-138-transfected HCT116 cells were shown. **P < 0.01 **(D and E)** Overexpression of miR-138 can inhibit the colony formation of CRC cell lines. Representative colony formation results for control-transfected and miR-138-transfected HCT116 cells were shown. **P < 0.01.

To explore the effect of overexpression of miR-138 on the tumorigenesis,motility and invasive capacity of CRC cells, transwell assays were performed. HCT116 cells transfected with miR-138 mimics or control microRNA were seeded into the chambers with or without matrigel, and their migratory and invasive potential were determined after 24 h, 36 h culture. Increased expression of miR-138 in HCT116 cells following transfection was confirmed via real-time PCR (Figure 2A). The results showed that the migratory capacity of HCT116 cells overexpressing miR-138 was reduced by 60.7% compared with the control groups (invasive capacity was reduced by 57.5%) (Figure 2B, C). We also evaluated the colony formation ability of HCT116 cells that had been transfected with miR-138 and or microRNA. The results showed that the miR-138-transfected cells formed considerably fewer and smaller colonies than control microRNA-transfected cells (Figure 2D), indicating a growth-inhibitory role of miR-138 in CRC cells (Figure 2D, E).

### TWIST2 was a direct target of miR-138 which inhibited its expression via binding to its 3′UTR

**Figure 3:**
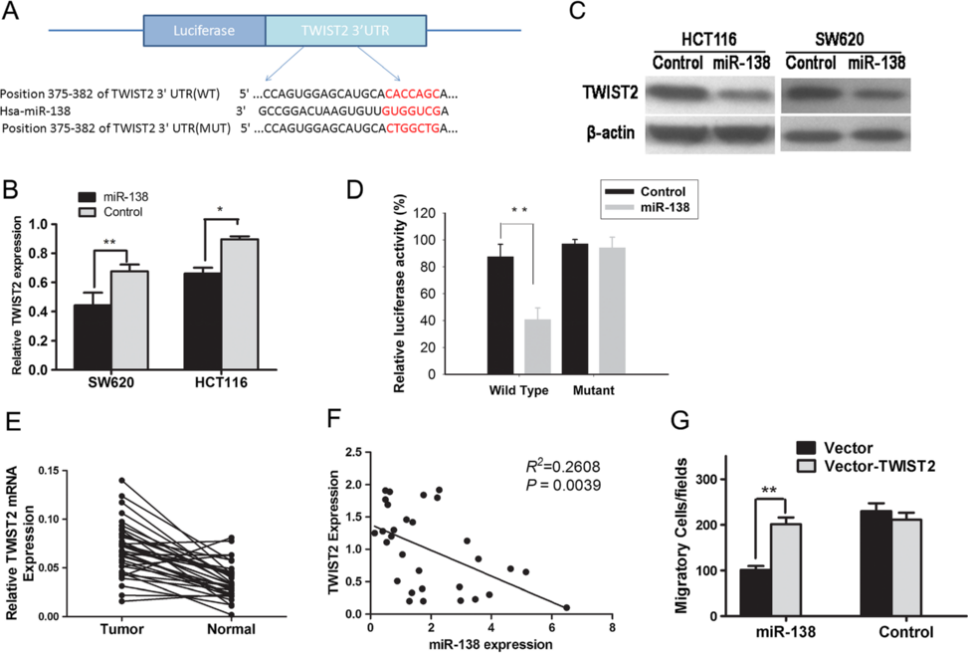
**TWIST2 is a direct functional target of miR-138. (A) **The conserved miR-138 binding sequence of TWIST2 or its mutated form was inserted into the C-terminal of the luciferase gene to generate pGL3-TWIST2-WT or pGL3-TWIST2-MUT, respectively. **(B)** Ectopic expression of miR-138 down-regulated the TWIST2 mRNA level in HCT116 cells, as determined by quantitative real-time PCR. **(C)** miR-138 decreased the TWIST2 protein level in HCT116 cells, as determined by Western Blot. The TWIST2 protein band intensities were quantified and normalized to β-actin intensities. **(D) **miR-138 targets the wild-type but not the mutant 3′UTR of TWIST2. miR-138 was cotransfected with pGL3-Control(the empty vector) or reporters constructed containing either wild-type or mutant TWIST2 3′UTR into 293 T cells. pGL3-Control was used as an internal control reporter. The luciferase activity assay was repeated three times. **P < 0.01. **(E)** Levels of TWIST2 mRNA is up-regulated in primary colorectal tumors compared with their adjacent normal tissues (n = 36). **(F)** Correlation between TWIST2 protein expression and miR-138 expression in CRC tissues. *P* = 0.0039, *R*^*2*^ = 0.2608. n = 36 **(G)** Rescue assays using HCT116 cells co-transfected with Vector-TWIST2 or Vector and miR-138 or Control. **P < 0.01.

It is accepted that miRNAs exert their function by regulating the expression of their downstream target genes. On the basis of two major prediction softwares, Targetscan (http://www.targetscan.org) and miRNA (http://www.microrna.org), we find that the 3′UTR of twist basic helix-loop-helix transcription factor 2 gene (TWIST2) contains a complementary site for the seed region of miR-138, and may be a potential target of miR-138 (Figure 3A). To validate the miRNA-target interaction and determine if miR-138 affects TWIST2 expression in the intracellular environment in CRC, the expression of TWIST2 was evaluated in HCT116 and SW620 cells following transfection with either miR-138 mimics or control microRNA. The transfection efficiency was determined by real-time RT-PCR (data not shown). Transfection of miR-138 resulted in significant reduction of TWIST2 mRNA (Figure 3B) and protein (Figure 3C) expression by real-time RT-PCR and Western blotting analysis, respectively. To further test the specific regulation through the predicted binding sites, we constructed a reporter vector consisting of the luciferase coding sequence followed by the 3′UTR of TWIST2 (wild type and mutant type) (Figure 3A). Cotransfection experiments showed that miR-138 decreased the luciferase activity of wild type for 45%, but this was not observed on mutant type (Figure 3D), indicating that TWIST2 is a direct target of miR-138 through binding to its 3′UTR.

### TWIST2 is up-regulated in primary CRC tissues and its expression correlates with miR-138 expression in CRC

TWIST2 was reported to be a potential oncogene in human cancers [15]. To corroborate the potential importance of TWIST2 in primary CRCs, we compared the level of TWIST2 expression in 36 paired tumors versus the surrounding normal tissues. The expression of the TWIST2 mRNA accessed by real-time PCR was significantly increased in CRC tumors by about 3 fold compared with the adjacent normal tissues (P < 0.001; Figure 3E). TWIST2 is overexpressed in 88.9% (32/36) of tumors compared with their normal counterparts (see Figure 3E).

To further evaluate the correlation between TWIST2 and miR-138 expression in primary CRCs, we compared the expression of TWIST2 and miR-138 in 36 primary CRCs. Expression of TWIST2 and miR-138 exhibited a significant inverse correlation (*P* = 0.0039, *R*^*2*^ = 0.2608; Figure 3F), further supporting the miR-138 target status of TWIST2.

### miR-138 inhibits CRC cell motility by targeting TWIST2

To determine whether TWIST2 is involved in the miR-138-induced inhibition of migration and invasion, we performed rescue assays. As shown in Figure 3G, concomitant overexpression of miR-138 and TWIST2 nullified the inhibitory effect of miR-138 on the cell migratory ability of HCT116 cells compared with cells transfected with miR-138 and the empty expression vector groups (*p* < 0.01). However, there was no significant difference between the group that co-transfection control microRNA with pcDNA3.1-TWIST2 and the group that co-transfection miR-138 with pcDNA3.1-TWIST2 (Figure 3G). These results demonstrated that miR-138 can inhibit CRC cell migration and invasion by targeting TWIST2.

### miR-138 inhibits CRC cell metastasis in vivo

**Figure 4:**
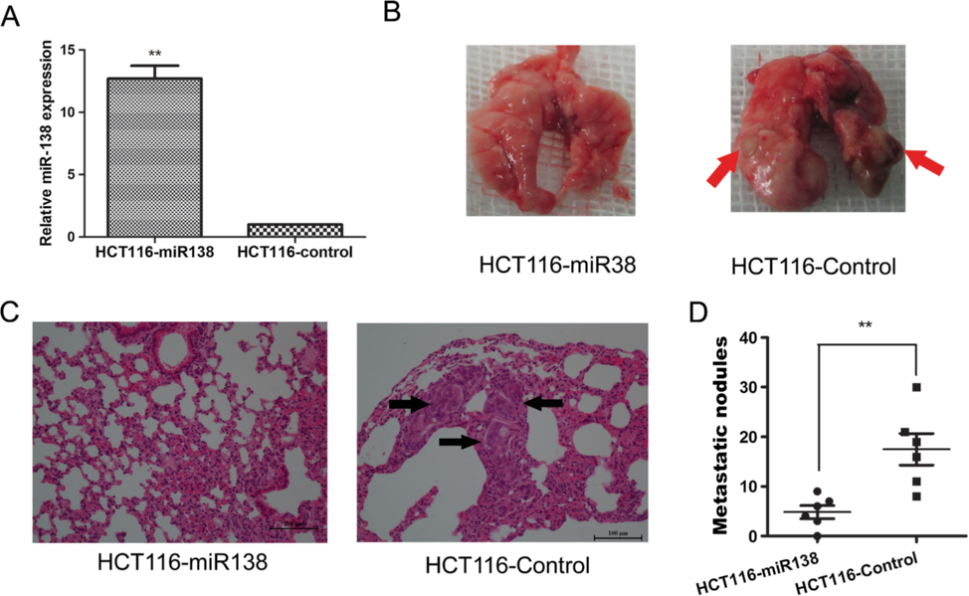
**Exogenetic expression of miR-138 suppresses CRC cell metastasis in vivo. (A)** Establishment of the stable cell lines overexpressing miR-138. The expression of miR-138 was markedly increased in the lentiviral vector-mediated miR-138-expressing stable cell line HCT116-miR138 compared with the control stable cell line HCT116-Control. **P < 0.05. **(B)** Representative lungs subjected to the indicated treatments. The metastatic nodules are indicated with arrows. **(C)** Representative results of hematoxylin and eosin staining of metastatic nodules in the lung are shown. Metastatic nodules are indicated with arrows. **(D)** Number of metastatic nodules in the lungs of mice. The nodules were examined under a microscope. **P < 0.01, n = 8.

To further investigate the role of miR-138 involved in CRC metastasis *in vivo*, we constructed a lentivirus vector to mediate the expression of miR-138 and established two stable cell lines, which were named HCT116-miR138 and HCT116-control (Figure 4A). Real-time PCR results showed that HCT116-miR138 cells expressed 13 fold miR-138 than control cells HCT116-control, indicating the construction was successful (Figure 4A). Then, we implanted HCT116-miR138 and HCT116-control cells into nude mice through the lateral tail vein respectively. As shown in Figure 4B, mice injected with HCT116-control cells formed larger metastates in the lungs compared with the HCT1116-miR138 cells (Figure 4B). The HE results showed that most of the mice injected with the HCT116-control cells displayed a large number of metastatic lung nodules after eleven weeks of growth. In contrast, injection of HCT116-miR138 cells generated fewer metastatic nodules than were observed in the controls (Figure 4C). And the results is statistical significance (Figure 4D).

### Correlations between miR-138 expressions and clinicopathological characteristics and prognosis

**Figure 5:**
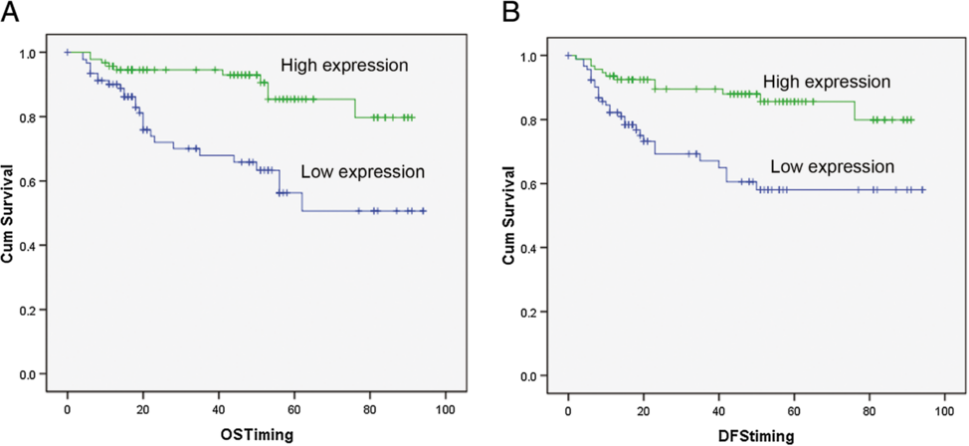
**Kaplan–Meier analysis of overall survival and disease-free survival for patients with miR-138 high or low expressions.** Overall survival (OS) **(A)** and disease-free survival (DFS) **(B)** curves for two groups defined by low and high expression of miR-138 in patients with CRC. The patients with low miR-138 expression had a significantly worse OS (*P* < 0.01) and DFS (*P* < 0.01) survival rate than those with high miR-138 expression.

To further investigate the clinicopathological and prognostic significance of miR-138 levels in patients with CRC, miR-138 levels were examined via real-time PCR in 187 tumor tissues and paired normal tissues. And miR-138 expression was divided into high or low expression group according to the median value. Therefore, patients with high or low miR-138 expression were 94 and 93, respectively. As shown in Table 1, no significant differences were observed between miR-138 expressions and age, sex, CEA level, CA199 level, tumor site, tumor Size, differentiation, and Local invasion. But, miR-138 expression is significant correlated with the patient’s lymph node metastasis and distant metastasis. Using Kaplan–Meier method and log-rank test, the overall survival (OS, Figure 5A) and disease-free survival (DFS, Figure 5B) of CRC patients with high miR-138 expression were both significantly longer than those with low miR-138 expression.

**Table 1:** The correlation between miR-138 expression and clinicopathological parameters of colorectal cancer

	**miR-138 expression**			
**Clinipathological parameters**	**Cases (n = 187)**	**Low (n = 93)**	**High (n = 94)**	***P*** *
Age (years)				
≤56	93	44(47.3%)	49(52.7%)	0.510
>56	94	49(52.1%)	45(47.9%)	
Sex				
male	104	55(52.9%)	49(47.1%)	0.335
female	83	38(45.8%)	45(54.2%)	
CEA Level				
0 ~ 5 ng/ml	99	40(40.4%)	59(59.6%)	0.428
>5 ng/ml	88	32(36.4%)	56(63.6%)	
CA199 Level				
0 ~ 35 u/ml	136	65(47.8%)	71(52.2%)	0.254
>35 u/ml	47	27(57.4%)	20(42.6%)	
Tumor Site				
Colon	101	47(46.5%)	54(53.5%)	0.343
Rectum	86	46(53.5%)	40(46.5%)	
Tumor Size (cm)				
≤5	122	60(49.2%)	62(50.8%)	0.466
>5	62	29(46.8%)	33(53.2%)	
Differentiation				
Well/Moderate	144	61(42.4%)	83(57.6%)	0.209
Poor	43	27(62.8%)	16(37.2%)	
pN				
0	109	37(33.9%)	72(66.1%)	0.000*
≥1	78	56(71.8%)	22(28.2%)	
pT				
T1 + T2	47	27(46.9%)	20(53.1%)	0.105
T3 + T4	141	79(56.0%)	62(44.0%)	
Distant metastasis				
M0	161	79(49.1%)	82(50.9%)	0.002*
M1	26	22(84.6%)	4(15.4%)	

*χ^2^ Test.

## Discussion

Metastasis is widely recognized as a poor prognostic factor in cancer. MicroRNAs have recently been demonstrated to potentially play a significant role in the progression of carcinogenesis [16]. To date, lots of different miRNAs regulate many known oncogenic and tumor suppressor pathways involved in the pathogenesis of CRC. For example, miR-192, miR-31 and miR-21 induce CRC cells resistance to 5-fluorouracil (5-FU) and may be important clinical markers of chemotherapy efficacy in colorectal cancer [17-19]. The miR-200 family suppresses the zinc finger E-box binding homeobox to promote CRC cell EMT and invasion [20]. In this work, we have started to shed light on the importance of miR-138 in CRC metastasis. While some studies have found miR-138 might be a potential tumor suppressor in some types of cancers, including ovarian cancer, head and neck squamous cell carcinoma, and cholangiocarcinoma [12,21,22]. In agreement with these reports showing the suppressor role of miR-138, we have also observed down-regulation of miR-138 in CRC tissues and cell lines. Restoration of miR-138 can reduce CRC cell colony formation, migration and invasion *in vitro*. Importantly, *in vivo* metastatic models revealed a significant decrease in tumor growth and metastatic capability treated with the stable cell lines which over-expressed miR-138. To our knowledge, this is the first study showing that miR-138 is down-regulated in CRC tissues and cell lines and overexpression of miR-138 can inhibit CRC migration and invasion *in vitro* and *in vivo*.

Previous studies indicated that miR-138 may have an effect on tumor metastasis by targeting SOX4 and Hif1a in ovarian cancer, MMP2/MMP9 in cholangiocarcinoma cells and FAK in hela cells [12,22,23]. In our study, we reported that TWIST2 is a direct target of miR-138. Overexpression of miR-138 can reduce CRC cell motility through targeting TWIST2. Meanwhile, we found that exogenous miR-138 expression or ablation of TWIST2 can both abrogate the metastasis of CRC cells and inhibition of miR-138 can promote the metastatic ability of CRC cells *in vitro* (data not shown). In addition, *in vivo* metastatic experiments confirmed that stable over-expression of miR-138 inhibited the metastatic ability of CRC cells.

To identify the putative miR-138 target genes, we applied combined in silico seed site analysis, Western Blot, and luciferase reporter assays. We demonstrated that TWIST2 is a direct target of miR-138, with the evidence that overexpression of miR-138 led to reduced luciferase activity with TWIST2 3′UTR and miR-138 can down-regulate TWIST2 mRNA and protein expression (Figure 3). Although the previous studies found that SOX4, Hif1a, and MMP2/MMP9 are all miR-138′s targets. In our study, we found that TWIST2 is a novel target of miR-138. We speculated that the same microRNA performs different functions through distinct target genes depending on the tissue or cell types.

Furthermore, we also noticed that overexpression of TWIST2 by the pcDNA3.1-TWIST2 plasmid nullifies the effect of suppression of cell invasion by miR-138. Noticeably, the proliferative and invasive capacity of TWIST2 overexpression alone is not significantly changed in CRC cells (data not shown). Knockdown of TWIST2 gene expression by siRNA had an effect on *in vitro* invasion capability that was similar to that of the restoration of miR-138. These properties support the notion that TWIST2 repression contributes to the tumor metastasis suppressive effects of miR-138. However, the suppressive effect of miR-138 on invasion is stronger than its effect in suppressing TWIST2 expression. This indicates that there might be additional miR-138 targets that act as tumor oncogenes. These findings suggest that miR-138 playing the tumor suppressive role is at least partly through decreasing the TWIST2 expression.

TWIST2 is a basic helix-loop-helix (bHLH) transcription factors have been implicated in cell lineage determination and differentiation. The protein encoded by this gene is a bHLH transcription factor and shares similarity with another bHLH transcription factor, Twist [24]. It was reported as an oncogene that correlates with poor prognosis in head and neck squamous cell carcinomas [25] and valuable prognostic marker for CRC [26]. And TWIST2 is also crucial for the EMT of cancer cells [15,27]. Here, we found that TWIST2 was overexpressed in CRC tumor tissues compared with adjacent normal tissues (Figure 3E). Enhanced TWIST2 expression was also observed in CRC cell lines (data not shown). And the expression of TWIST2 and miR-138 in CRC tissues is inversely correlated (Figure 3F). These results inferred that down-regulation of miR-138 endows the metastatic potential to CRC cells, promotes the metastasis of CRC. And we speculate that miR-138 may function as a tumor metastasis inhibitor through the inhibition of CRC EMT by targeting TWIST2.

Dysregulated miRNA signatures have been associated with clinical outcome [28]. The potential clinical application of miR-138 in human cancer, especially in CRC has never been reported. In this study, we extended our analyses by assaying miR-138 expression in specimens from 187 human CRC patients. Low-level miR-138 expression was found to be significantly associated with a more aggressive tumor phenotype and high preoperative CEA levels and was also shown to be a strong independent predictor of a short disease-free survival period and short overall survival for patients with CRC.

## Conclusion

We have identified that miR-138 is down-regulated in CRC. miR-138 suppressed CRC cell migration and invasion, at least in part via inhibition of oncogene TWIST2. And miR-138 may be a candidate tumor suppressive miRNA of CRC and might be a new prognostic biomarker for CRC patients.

## Abbreviations

CRC: Colorectal cancer; TWIST2: Twist basic helix-loop-helix transcription factor 2; microRNA: miRNA; IHC: Immunohistochemistry; qPCR: Quantitative polymerase chain reaction; 3′UTR: 3′-Untranslated region; EMT: Epithelial-mesenchymal transition.

## Competing interests

The authors declare that they have no competing interests.

## Authors’ contributions

LML and GQH were the major players in the experimentation, data collection, preparation of results, writing and statistical analysis. HYZ and JRH were involved in the sample collection. YHG and YSL performed the animal experiments. JRH was the principal investigator who had designed the project, organized experimental materials and supervised experimental work. All authors read and approved the final manuscript.
